# J-ACCESS investigation and nuclear cardiology in Japan: implications for heart failure

**DOI:** 10.1007/s12149-023-01836-x

**Published:** 2023-04-11

**Authors:** Kenichi Nakajima, Tsunehiko Nishimura

**Affiliations:** 1 Department of Functional Imaging and Artificial Intelligence, Kanazawa University, Kanazawa, 920-8640 Japan; 2 Graduate School of Medical Science, Kyoto Prefectural University of Medicine, Kyoto, Japan

**Keywords:** Coronary artery disease, Myocardial perfusion imaging, Heart failure, Risk, Multicenter

## Abstract

While coronary heart disease remains a global cause of mortality, the prevalence of heart failure (HF) is increasing in developed countries including Japan. The continuously increasing aging population and the relatively low incidence of ischemic origins are features of the HF background in Japan. Information about nuclear cardiology practice and prognosis has accumulated, thanks to the multicenter prognostic J-ACCESS investigations (Series 1‒4) over two decades in Japan. Although the rate of hard cardiac events is lower in Japan than in the USA and Europe, similar predictors have been identified as causes of major adverse cardiac events. The highest proportion (50–75%) of major events among patients indicated for nuclear cardiology examinations in the J-ACCESS registries is severe HF requiring hospitalization. Therefore, the background and the possible reasons for the higher proportion of severe HF events in Japan require clarification. Combinations of age, myocardial perfusion defects, left ventricular dysfunction, and comorbid diabetes and chronic kidney disease are major predictors of cardiovascular events including severe HF. Although the Japanese Circulation Society has updated its clinical guidelines to incorporate non-invasive imaging modalities for diagnosing chronic coronary artery disease, the importance of risk-based approaches to optimal medical therapy and coronary revascularization is emphasized herein.

## Coronary artery disease (CAD) and heart failure (HF) in Japan

**Fig. 1:**
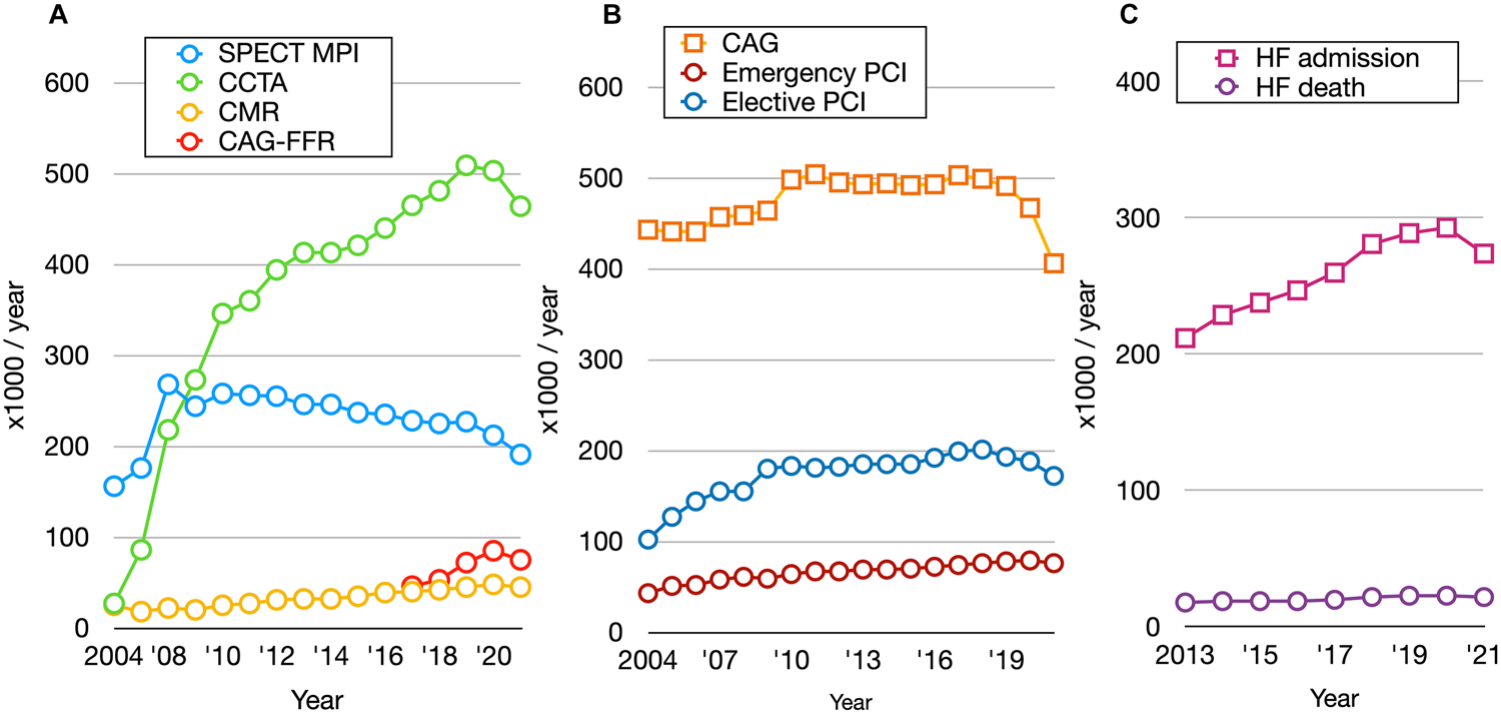
Annual frequencies of clinical nuclear imaging and coronary revascularization (2004‒2021), and heart failure admission and death (2013‒2021) in Japan. Panel **A** SPECT MPI, CCTA, CMR and FFR during CAG. Panel **B** CAG, emergency, and elective PCI. Panel **C** Hospital admissions for HF and deaths due to HF during admission. *CAG* coronary angiography, *CCTA* coronary computed tomography angiography, *CMR* cardiac magnetic resonance imaging, *FFR* fractional frow reserve measurements, *HF* heart failure, *MPI* myocardial perfusion imaging *PCI* percutaneous coronary intervention

Coronary artery disease (CAD) remains the leading cause of mortality and morbidity in developed countries, including Japan. The global prevalence of HF is increasing despite advances in pharmacological and non-pharmacological therapies, and early recognition and appropriate treatments are recognized as major health-related issues. The number of patients aged > 20 years with HF is 6.5 million in the USA, and > 1.2 million in Japan with HF are outpatients [1]. The Japanese Registry of All Cardiac and Vascular Diseases (JROAD) statistics generated by the Japan Circulation Society (https://www.j-circ.or.jp/jittai_chosa/about/report/https://www.j-circ.or.jp/jittai_chosa/about/report/, accessed on December 2022) indicate that ~ 0.3 million patients were diagnosed and hospitalized with HF in Japan during 2020 (Fig. 1). The prevalence of HF among persons aged ≥ 20 years in the USA was 2.1% (5.1 million) during 2010 [2], when the estimated prevalence in Japan was 1%. However, the population of people aged > 75 years is increasing and is predicted to reach 20% by 2025. In fact, statistics from the Japanese Registry of Acute Decompensated Heart Failure (JROADHF) show that the mean age of 13,238 patients at 128 hospitals was 78.0 ± 12.5 years, with those aged > 75 years accounting for 68.9%. During a median follow-up of 4.3 years, the respective rates of cardiovascular death and the re-hospitalization for HF were 7.1 and 21.1 per 100 person-years [3]. The relatively low incidence (26.6%) of ischemic etiology in this multicenter registry is characteristic of Japanese patients with HF, but the proportion of HF caused by ischemic heart disease has recently increased. In contrast, the proportion of ischemic etiology in the European Society of Cardiology HF long-term (ESC-HF-LT) registry is 56.5% [4, 5]. Thus, we anticipate a rapid increase in the numbers of patients with HF, which will also increase the burden on the Japanese healthcare system. Moreover, the social frailty of super-aged elderly and the increasing proportions of individuals with dementia are important targets in Japan [6]. The rate of pump-failure death is high in aged populations with HF. Moreover, HF with preserved ejection fraction (HFpEF) might have contributed to 16–50% of HF according to recent databases [1, 3, 7], and hypertension and ischemic heart disease are major underlying diseases in Japan. Strategies for avoiding sudden cardiac death could also be an important target in younger patients. The Chronic Heart Failure Analysis and Registry in the Tohoku District (CHART) studies 1 and 2 have identified westernized ischemic etiology and clinical characteristics in Japan as increased comorbidity and HF admissions [8].

An overview of imaging modality statistics in JROAD showed that the rate of coronary CT angiography (CCTA) evaluations has rapidly increased, whereas that of myocardial perfusion imaging (MPI) has gradually decreased over the past decade (Fig. 1). Coronary angiography (CAG) and elective percutaneous coronary interventions (PCI) have slightly decreased since 2018, which was partly due to changes in reimbursement policies that are discussed in the section entitled Guidelines for stable CAD updated by the Japanese Circulation Society (JCS) below.

With respect to nuclear cardiology practice, few multi-center registries in Japan include patients with CAD and HF, and the prospective prognostic study of Japanese Assessment of Cardiac Events and Survival Study by Quantitative Gated SPECT (J-ACCESS) 1‒4 are important databases for understanding the role of MPI [9–13]. Although J-ACCESS includes patients with definite or suspected CAD and investigations of HF outcomes were not intended, the most prevalent outcome was severe HF requiring hospitalization. Therefore, the incidence and the reasons for HF events in patients who underwent nuclear studies require clarification. This article reviews J-ACCESS investigations conducted since 2001 to the present, as well as recent Japanese trends in nuclear cardiology, and discusses possible roles of nuclear imaging with a focus on HF.

## J-ACCESS and prognosis of ischemic heart disease in Japan

**Fig. 2:**
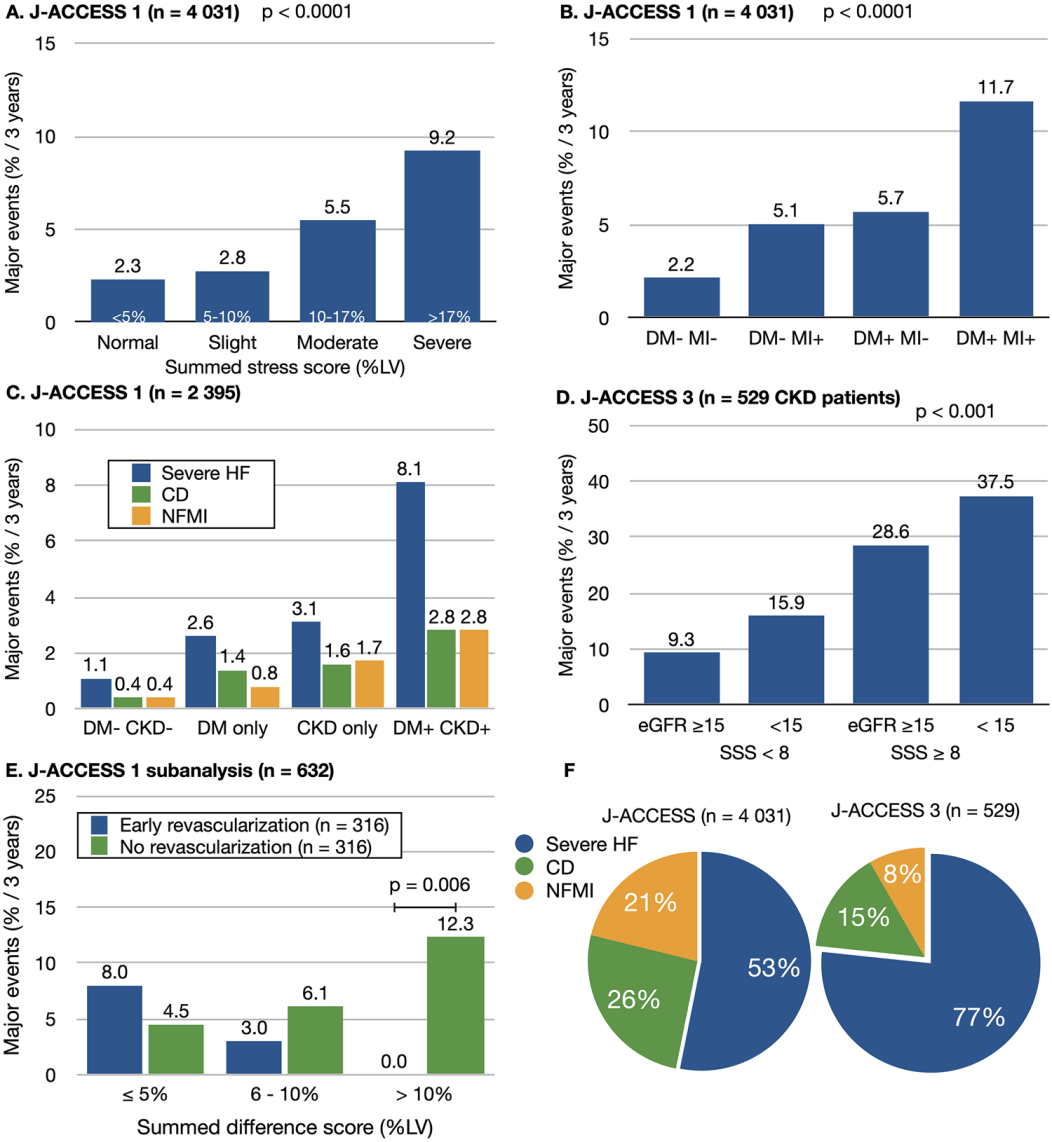
Results of J-ACCESS investigations. All results are presented as major event rates for 3 years. **A** Summed stress score (%LV); **B** DM and prior MI; **C** DM and CKD; **D** eGFR and SSS; **E** Major events in groups with early (< 60 days after myocardial perfusion study) and no revascularization; **F** Breakdown of major events: CD, non-fatal MI, and severe HF requiring hospitalization. All graphs were created using original J-ACCESS database [9, 12, 23, 27]. *CD* cardiac death, *CKD* chronic kidney disease, *DM* diabetes mellitus, *eGFR* glomerular filtration rate, *HF* heart failure, *LV* left ventricle, *MI* myocardial infarction, *SSS* summed stress score

Epidemiological and race differences between Japan and the USA as well as European countries should be considered when evaluating prognoses associated with MPI. However, such a database was not available in Japan during the year 2000. We therefore created the first nationwide database that specifically focused on evaluations by quantitative myocardial perfusion single-photon emission computed tomography (SPECT), namely the Japanese Assessment of Cardiac Events and Survival Study by Quantitative Gated SPECT (J-ACCESS; QGS; Mount Sinai Medical Center, Los Angeles, CA, USA) [9]. Four series of J-ACCESS investigations over the next two decades focused on different types of targeted patients (Table 1, Fig. 2).

**Table 1:** Background and major events in J-ACCESS investigations

	J-ACCESS	J-ACCESS 2	J-ACCESS 3	J-ACCESS 4
Registered patients (*n*)	4629	513	549	494
Registered patients analyzed (*n*)	4031	485	529	298
Hospitals (*n*)	117	50	62	59
Disease	CAD	Type 2 diabetes	CKD	CAD before revascularization
Follow-up interval (years)	3	3	3	1
Age (years)	66 ± 11	67 ± 8	72 ± 11	70 ± 9
Summed stress score	8.7 ± 11.2	2.8 ± 4.6	1.9 ± 3.8	10.7 ± 6.1
LVEF (%)	62 ± 14	67 ± 10	62 ± 15	68 ± 11
Diabetes mellitus	29%	100%	42%	47%
eGFR (mL/min/1.73 m^2^)	67 ± 31 (*n* = 2395)	82 ± 28	29 ± 13	63 ± 17
Endpoint of major events	CD, NFMI, severe HF	Cardiovascular events	CD, NFMI, severe HF	CD, NFMI, severe HF
Major events (1 + 2 + 3)	189	17	60	4
Cardiac death (1) (sudden cardiac death included)	57 (1.4%)	5 (1.0%)	9 (1.7%)	0 (0.0%)
NFMI (2)	39 (1.0%)	9 (1.9%)	5 (0.9%)	1 (0.3%)
Severe HF (3)	93 (2.3%)	3 (0.6%)	46 (8.7%)	3 (1.0%)
Severe HF/major events	49.2%	17.6%	76.7%	75%
Other events		Cerebrovascular TIA (*n* = 17)		

Data are shown as means ± standard deviation, and numbers with or without ratios (%)

*CAD* coronary artery disease, *CD* cardiac death, *CKD* chronic kidney disease, *HF* heart failure, *NFMI* non-fatal myocardial infarction, *TIA* transient ischemic attack

The J-ACCESS registered 4629 consecutive patients with confirmed or suspected CAD from 117 institutions in 2001. All patients underwent ^99m^Tc-tetrofosmin stress-rest studies and quantitative perfusion defect scoring with summed stress/rest/difference scores (SSS/SRS/SDS) and quantitative left ventricular functional analysis [14]. The major events were cardiac death, non-fatal myocardial infarction (MI), and severe HF requiring hospitalization. These patients were followed up for 3 years during the J-ACCESS investigations 1‒3, when 175 (4.3%) patients developed major cardiac events, of which half were severe HF requiring hospitalization (Fig. 2A,B,C). Although the rate of major events was lower in Japan than in the USA between 1998 and 2003 [15, 16], the major predictors of events were similar among several multicenter studies. For example, normal myocardial perfusion SPECT with normal ventricular function indicated a good prognosis [17, 18]. Myocardial perfusion defects at stress and a lower left ventricular ejection fraction (LVEF) were major predictors of cardiac events. Having comorbid diabetes mellitus or chronic kidney disease (CKD) increased major event risk ~ twofold, which was less fatal but similar to findings in the USA [19–21]. Perfusion abnormalities revealed by coronary angiography (CAG) had significant additive value over coronary stenosis for predicting cardiac events [22]. A propensity-score-matched analysis of early revascularization and medications revealed that such abnormalities were effective for patients with > 10% ischemia (Fig. 2E) [23]. While the study included a high proportion of serious HF events (49‒77%; J-ACCESS-1, 3 and 4), CKD, greater SSS, and higher end-systolic volume (ESV) or low LVEF were independent and additive predictors of refractory HF risk in patients with confirmed or suspected CAD [24].

The J-ACCESS 2 study investigated the prognosis of patients with type-2 diabetes and asymptomatic CAD [11]. Abnormal perfusion findings indicated myocardial ischemia and/or scar in 32% of 485 patients. Five (1.0%) of 485 patients developed fatal cardiovascular events, and severe HF and other events comprised mostly non-fatal acute coronary syndrome, new onset of stable angina pectoris, and cerebrovascular accidents in 9 (1.9%), 10 (2.1%), and 15 (3.1%) of these patients, respectively, during a 3-year follow-up. Multivariate analysis revealed that SSS ≥ 9, low eGFR, and currently smoking were significant variables, indicating that this group of patients needed active treatment strategies.

The J-ACCESS 3 prospective cohort study of CKD investigated predictions of cardiac events among patients with eGFR < 50 mL/min/1.73 m^2^ without definitive CAD [11]. The event-free survival rate was lower among patients with renal dysfunction and a higher SSS (Fig. 2D). A C-reactive protein value of ≥ 0.3 mg/dL independently predicted cardiac events, suggesting that this additional inflammatory parameter is important as a pathophysiological basis for developing cardiovascular events. The highest fraction of the events consisted of severe HF (77%), which confirmed the importance of therapeutic intervention for patients with CKD (Fig. 2F).

The J-ACCESS 4 study investigated patients with coronary revascularization [13]. Although cardiology guidelines describe myocardial ischemia as an indication for coronary revascularization in patients with chronic stable CAD, whether myocardial ischemia could be a target of coronary revascularization to reduce cardiac events was not verified in a Japanese population. When patients were classified into two groups with a threshold of 5% ischemic reduction, the prognosis of those with ≥ 5% improvement after revascularization was significantly better, and essentially agreed with the findings of a large cohort in the COURAGE study [25].

The J-ACCESS series revealed a lower prevalence of CAD and a better prognosis for Japanese patients than those in other developed countries. The hard cardiac event rate of Japanese patients was < 3% over 3 years, which was less than half that of patients in the USA [9, 10, 16].

## J-ACCESS risk model for stratifying prognosis in patients with suspected CAD

**Fig. 3:**
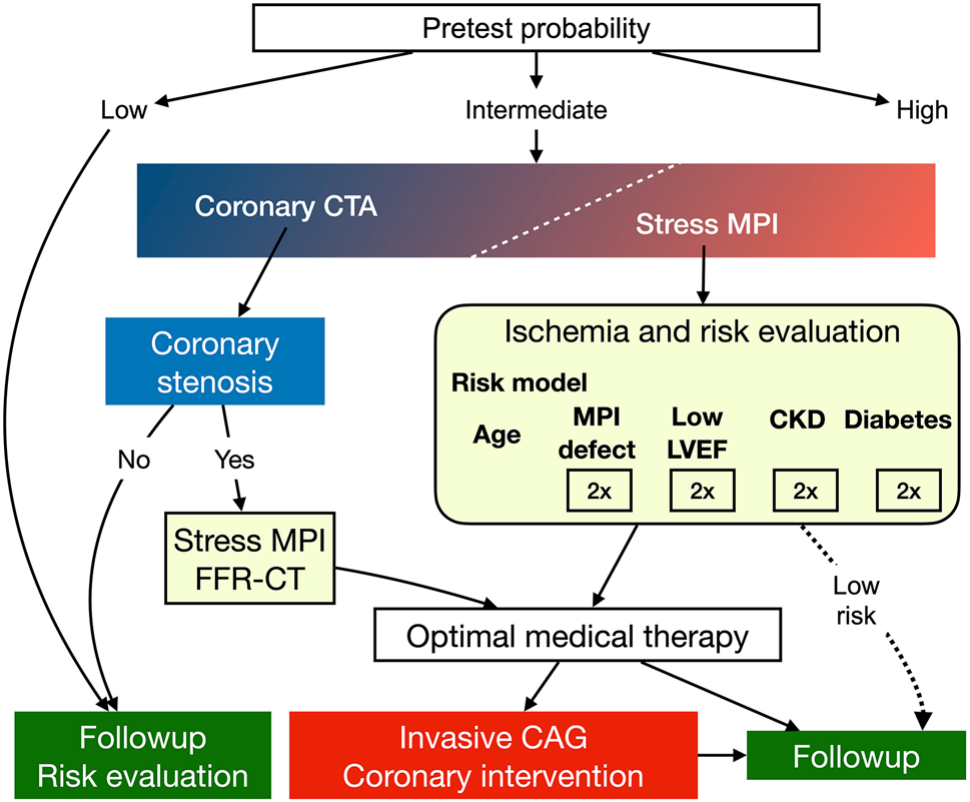
Roles of non-invasive imaging including coronary CT angiography (CTA) and stress myocardial perfusion imaging (MPI). Possible role of risk evaluation is included to indicate follow-up, coronary angiography (CAG) and if necessary, coronary intervention. Major cardiac event risk was doubled by each factor and is indicated as 2 × . *CKD* chronic kidney disease, *FFR-CT* fractional flow reserve measurement based on X-ray CT, *LVEF* left ventricular ejection fraction

Appropriate risk stratification is a major target of MPI studies because total risk to patients can be determined by comorbidities in addition to perfusion and function. We therefore proposed a risk chart or software based on statistical multivariable models derived from the J-ACCESS results. The risk model included the variables of age, SSS, LVEF, and diabetes in the initial model, then was revised by adding eGFR as a variable [26, 27]. The risk chart and Heart Risk View software (Nihon Medi Physics, Tokyo, Japan) have been incorporated into current clinical practice. The risk model calculates major event rates as ratios (%) per 3 years. This model was intended for application to patients with suspected CAD and a background similar to that in the J-ACCESS study [28]. We validated the model by comparing predicted and actual outcomes between a new patient cohort in the APPROACH study [29] and patients in J-ACCESS 3 [30]. The results showed that the risk model effectively stratified risk, whereas actual event rates were equally high across all risk groups among patients with eGFR < 15 mL/min/1.73 m^2^. The model was not intended simply to numerically report event rates. More importantly, risk stratification incorporating MPI and clinical variables allowed the classification of patients at low, intermediate, and high risk for cardiovascular events. This provided useful information upon which to base decisions regarding medications and other types of therapies such as coronary revascularization (Fig. 3).

Although risk can be evaluated by stress MPI, how prognostic indices can be applied in clinical practice is more important [31]. Even when degrees of ischemia are similar, event rates will be higher in patients with, than without comorbid diabetes, CKD and a reduced LVEF. This might have a practical value when considering invasive therapeutic strategies.

**Fig. 4:**
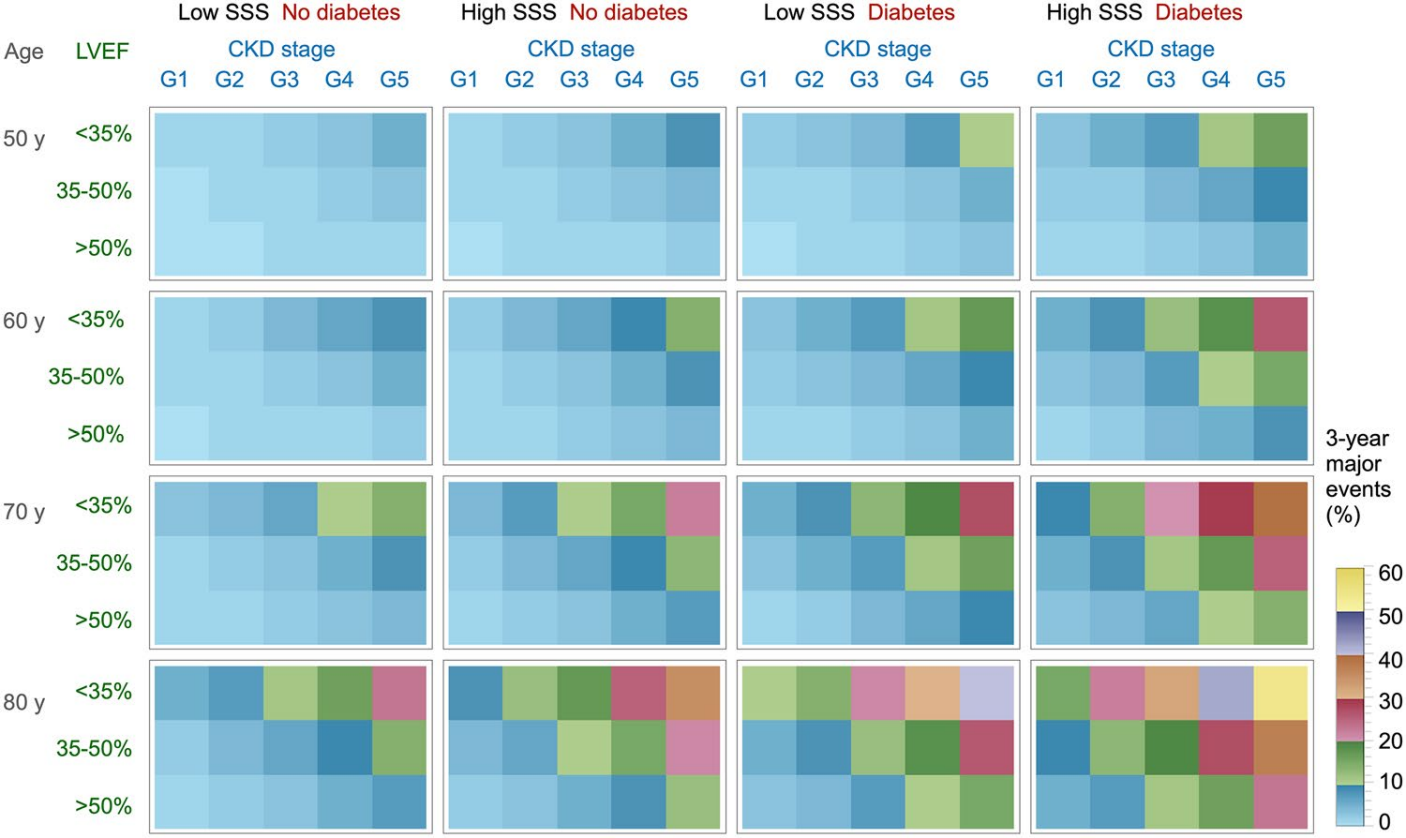
Risk levels estimated based on age, LVEF, stress perfusion defects, and CKD stages G1‒G5. Heatmap shows risk levels for predicting 3-year major cardiac events. Color scale represents 0–60%. *CKD* chronic kidney disease, *LVEF* left ventricular ejection fraction

Table 2 shows multivariable logistic analyses recalculated to predict major cardiac events using the continuous variable of age, and the categorical variables of LVEF (< 35%, 35‒50%, > 50%), eGFR stage (G1, ≥ 90; G2, 60‒89; G3, 30‒59; G4, 15‒29; G5, < 15 mL/min/1.73 m^2^), diabetes (0, 1), and SSS grades (0, 1, 2, and 3: scores < 4; 4‒8; 9‒12 and > 12, respectively). This table was recalculated based on previous information [27] using the same categorical variables except for age. The model structure revealed how each variable affects major event outcomes. Combining moderate-to-high SSS, low eGFR, low LVEF, and diabetes synergistically doubled the calculated risk (Fig. 3). Figure 4 shows levels of risk for major cardiac events estimated by the model to understand the contribution of each factor. Since the model included severe HF as a major event, we created another risk model, namely cardiac death and non-fatal MI for estimating hard events. Diagnostic use of multivariable risk model incorporating SSS, left ventricular volume, diabetes, hypertension, and number of risk factors has also been proposed for the diagnosis of multivessel CAD and indications for stress-only imaging [32]. A combination of diabetes and CKD offers important predictors when considering the background of Japanese patients with HF, because the JROAD HF statistics revealed that 30.1% and 38.9% of patients, respectively, had comorbid diabetes and CKD [3].

**Table 2:** Multivariable analyses to predict major and hard cardiac events

Term	Estimate	SE	*χ*^2^	OR	Lower 95%	Upper 95%	*p*
Major cardiac events							
Intercept	− 9.979	0.96	108				
Diabetes (0, 1)	0.793	0.201	15.7	2.21	1.49	3.28	< 0.0001
eGFR stages 1‒5	0.465	0.114	16.5	1.59	1.27	1.98	< 0.0001
SSS grades 0‒3	0.272	0.089	9.3	1.31	1.10	1.56	0.0023
EF grades (1‒3)	0.671	0.157	18.3	1.96	1.43	2.26	< 0.0001
> 50, 35‒50, < 35							
Age (years)	0.060	0.013	21.9	1.06	1.04	1.09	< 0.0001
Hard cardiac events							
Intercept	− 9.606	1.329	52.2				
Diabetes (0,1)	0.733	0.286	6.6	2.08	1.19	3.66	0.0103
eGFR Stages 1‒5	0.643	0.15	18.5	1.9	1.41	2.53	< 0.0001
SSS grades 0‒3	0.239	0.11	4.7	1.27	1.02	1.58	0.0297
Age (years)	0.051	0.018	7.8	1.05	1.02	1.09	0.0053

Hard events: cardiac death, non-fatal myocardial infarction; major events: cardiac death, non-fatal myocardial infarction, and severe heart failure requiring hospitalization

*eGFR* estimated glomerular filtration rate, *OR* odds ratio, *SE* standard error, *SSS* summed stress score

The applicability of such a risk model requires careful consideration, as it is not universally applicable to diverse populations. The backgrounds of patients upon which to create the model, contemporary medications and interventions, comorbidities, healthcare systems, and many other factors affect predictive models. Our model was applicable to Japan, and an Italian prognostic study found its direct use limited [33]. However, the principal ideas including major variables, such as age, myocardial perfusion or extent of ischemia, LV function, and comorbid diabetes, and CKD fit Japanese patients might be acceptable choices of predictors in general.

## Guidelines for stable CAD updated by the Japanese circulation society (JCS)

The ISCHEMIA trial highlighted the effectiveness of a contemporary optimal medical therapy (OMT) strategy including risk factor modification to prevent serious cardiac events [34, 35]. The COURAGE study also found that adding coronary revascularization to OMT did not necessarily result in a prognostic benefit, whereas patients with ≥ 5% reduction in myocardial ischemia had lower risk for death and myocardial infarction irrespective of treatment strategies [25]. These new perspectives also affected Japanese clinical practice, and the JCS suggested strategic algorithm [36], whereas the prevalence of CCTA evaluations has rapidly increased. The updated guidelines incorporated strategies starting from pre-test probability and a clinical likelihood of CAD to guide downstream non-invasive tests for stable CAD. Patients with intermediate-to-high pre-test probability can undergo further non-invasive imaging modalities, including CCTA and stress imaging. When both CCTA and stress imaging are available, CCTA is preferred to rule out CAD, whereas functional stress imaging is preferred as an initial screen of patients with a high pre-test probability or known history of CAD for risk assessment. The approaches of CCTA and stress imaging are typically summarized as rule-out and rule-in strategies, respectively. After post-test risk assessment, OMT is generally applied in the next step, and a decision to proceed with invasive coronary angiography will be reached for patients with uncontrolled angina despite OMT, high-risk CAD such as left main CAD or its equivalent, inconclusive non-invasive imaging tests, and HF with suspected CAD. Measurements of fractional flow reserve (FFR) are also important to determine indications of PCI aimed at reducing cardiovascular events.

The prospective, multicenter Japanese CVIT-DEFER registry that included 3,228 of 3,804 consecutive patients with angiographically moderate lesions of the coronary artery found frequent mismatches between angiographic stenosis and FFR, suggesting the clinical importance of physiological assessments to guide PCI [37]. Japanese reimbursement for elective PCI in patients with CAD was updated in 2018 to include a need for functionally proven ischemia, which slightly decreased the prevalence of CAG and elective PCI (Fig. 1). Considering the recent rapid increase in the numbers of patients undergoing CCTA in Japan, a CCTA-first strategy might prevail. Nonetheless, the increase in aged patients with multiple comorbidities and the recommendations for physiology-based PCI support the application of nuclear cardiology approaches. Not simply taking a dogmatic CT-first approach is an important viewpoint. The most appropriate test should be selected based on the findings of careful clinical evaluation to ensure the “right test for the right patient” [38]. The patient-first approach is the key to clinical practice in an era when multimodal imaging has become part of a widespread clinical workup.

Types of coronary revascularization, namely PCI and CABG, and medications have also become important topics. Ten-year data from the STICH studies have associated lower all-cause mortality with surgical revascularization compared with medical therapy [39]. The JROAD statistics (2021) revealed ~ 76,000 and ~ 172,000 emergency and elective PCIs, respectively, as well as ~ 9100 (on pump) and 7300 (off pump) coronary artery bypass surgeries (CABGs). The ratio of CABG to PCI was 6.6%. In contrast, trends in coronary revascularization in the USA revealed a CABG-to-PCI ratio of 39%, while the numbers of both PCIs and CABGs decreased between 2003 and 2016 [40]. A comparison of FFR-guided PCI and CABG in the FAME-3 investigation of three-vessel disease showed that PCI was not non-inferior to CABG with respect to the incidence of a composite of death, MI, stroke, or repeat revascularization at one year [41]. The CREDO-Kyoto PCI/CABG registry Cohort-3 (14,927 patients; follow-up, 5.7 years) assessed all-cause and cardiovascular death among patients with three-vessel disease [42]. That study associated PCI with a significantly higher risk for all-cause death than CABG, whereas risk for cardiovascular death among those treated by PCI did not exceed CABG in the real-world clinical era of drug-eluting stents in Japan. While stent technology and materials have improved, coronary artery patency might be prolonged with a less invasive approach such as CABG. The indications for CABG are therefore moving to more complex coronary lesions, reduced left ventricular function, and complications of diabetes and/or CKD.

## Prediction of new-onset HF

While risk for major cardiac event rates were analyzed, further investigation focused more specifically on HF in a subset of the J-ACCESS database [24]. Among 3835 patients with confirmed or suspected CAD, 71 required aggressive medical treatment due to new-onset congestive HF symptoms for 3 years. Multivariable Cox hazard models revealed that CKD, the ESV index, and moderate to high SSS predicted refractory HF (Table 3). This combination of factors had the greatest prognostic value compared with single or combined variables. The reasons for the high event rate in CKD might be attributed to fluid retention, myocardial stiffness and endothelial dysfunction in arterioles, none of which is estimated by anatomical studies using invasive and coronary CT angiography. One of the major factors for predicting HF was SSS.

**Table 3:** Multivariable Cox proportional hazard model for new-onset heart failure events Adapted from [24]

Variables	Wald *χ*^2^	HR	95% CI	*p*
Total patients (*n* = 3835)				
Chronic renal dysfunction*	22.4	6.23	2.92–13.28	< 0.0001
ESVI at rest (mL/m^2^)	18.3	1.02	1.01–1.03	< 0.0001
SSS (high vs. low)^†^	16.1	3.01	1.76–5.18	< 0.0001
Subset of patients excluding 276 who were treated by coronary revascularization				
Age (years)	31.5	1.11	1.07–1.15	< 0.0001
LVEF (%)	6.1	0.96	0.93–0.99	0.0133
ESVI at rest (mL/m^2^)	5.4	1.02	1.00–1.03	0.0207

*CI* confidence interval, *ESVI* end systolic volume index (mL/m^2^), *HR* hazard ratio, *LVEF* left ventricular ejection fraction, *SSS* summed stress score.

*Serum creatinine 1.2 and 1.0 mg/mL for men for women, respectively, or estimated glomerular filtration rate < 60 mL/min/1.73 m^2^ at baseline

^†^SSS, high > 8/80 (> 10% of left ventricle); low, 0–8 (≤ 10% of left ventricle)

The J-ACCESS studies did not select SDS as a major variable to predict events, probably due to relatively small ischemic lesions and a relatively small proportion of acute coronary syndrome after entry. In addition, patients with early coronary revascularization within the first 60 days were excluded from the analysis of prognosis because the choice of revascularization might have been influenced by SPECT diagnoses at the time. The ESV was also a reasonable variable showing that dilated left ventricular volume and a low LVEF could predict HF events.

One-third to half of patients with HF has a background of HFpEF. However, HFpEF was not investigated using variables such as diastolic dysfunction determined by gated SPECT and/or echocardiography in the J-ACCESS study. The mean LVEF was higher and the prevalence of HF with reduced EF was lower in the JROADHF, than in other registries, while higher mortality rates in that registry suggested a relatively higher prevalence of HFpEF partly due to the more advanced age of the patients [3, 4]. A recent meta-analysis has associated diabetes with new-onset HF especially in young populations and recurrent HF particularly in women or individuals with HFpEF [43]. The contributions of risk factors, such as sex, aging, hypertension, and diabetes to systolic and diastolic functions, and actual prognostic impact require further evaluation of Japanese patients with HF.

## Roles of ^123^I-labeled radiopharmaceuticals in CAD and HF

The situation in Japan regarding the use of ^123^I-labeled radiopharmaceuticals, such as ^123^I-beta-methyliodophenylpentadecanoic acid (BMIPP) and ^123^I-metaiodobenzylguanidine (MIBG), for patients with CAD and HF is unique. These ^123^I-labeled radiopharmaceutical tracers are included in 20% of nuclear cardiology studies, and they have been available since the 1990s [44]. Current JCS clinical guidelines for Diagnosis of Chronic Coronary Heart Diseases were issued by the JCS in Japanese and English during 2018 and 2021, respectively [45]. They recommend BMIPP imaging for myocardial ischemia and risk/prognosis (Classes I‒IIa). Myocardial sympathetic imaging is also recognized in the prognostic evaluation of HF and ventricular tachyarrhythmias. A Japanese pooled ^123^I-MIBG database has verified powerful prognostic values for patients with chronic HF [46, 47]. Defect scores for ^123^I-BMIPP indicate possible risk stratification in patients with acute and chronic HF [48, 49]. Since these ^123^I radiopharmaceuticals have not been approved outside Japan, their clinical role might have been universally underestimated. However, HF has been investigated in patients by innervation imaging using ^123^I-MIBG for research purposes in the USA and Europe [50]. The possibility of optimizing patient selection for evaluation using expensive cardiac devices to determine lethal cardiac arrhythmias has also been investigated. The effectiveness of MIBG indices for predicting arrhythmic and overall mortality risk and the possible integration of the clinical variables into the prediction model require further investigation and refinement for clinical practice [51, 52]. Simultaneous assessment of myocardial perfusion and innervation in patients with HF using contemporary semiconductor cadmium–zinc–telluride (CZT) detectors might also become an option with reduced radiation exposure [53]. Large-scale studies are mandatory to support the clinical application of ^123^I tracers as a useful adjunct to acquiring perfusion images of patients with CAD and HF.

## Limitations

The J-ACCESS studies conducted between 2001 and 2014 have several limitations. The most recent advances in technology and quantitation, such as integrated attenuation and scatter correction, and CZT detectors that allow reduced administered doses were not included. Treatment strategies for CAD and HF have been updated during the past two decades although appropriate contemporary strategies were applied during all J-ACCESS studies. The results cannot be applied to patients with valvular heart disease, and dilated, hypertrophic, and secondary cardiomyopathies, as they were excluded from the multicenter registry. Patients with severe HF were also not included at the timing of registration, although we discussed new-onset of HF retrospectively in this article. Moreover, recent advances in imaging technology and analytics, such as radiomics and artificial intelligence, or machine learning might offer novel strategies in cardiology, neurology, and oncology [52, 54–57]. Although a new patient cohort has not been included in the J-ACCESS registry, the application of artificial intelligence to quantify perfusion defects and/or ischemia is under investigation using current image databases.

## Conclusions

The J-ACCESS investigations (series 1‒4) established a nationwide prognostic database of Japanese patients from 2001 to the present. An overview of current trends in HF indicated that a high proportion of HF among major cardiac events in J-ACCESS might be associated with increasing numbers of elderly persons, a high fraction of HFpEF, and a higher frequency of non-ischemic etiology in Japanese patients with HF. The predictors of major adverse events including hospitalization for severe HF are age, baseline left ventricular function, myocardial perfusion defects, and comorbid diabetes and CKD. Thus, comprehensive risk-based approaches using appropriate multicenter databases are required for groups of patients with CAD and HF. While changes in strategic algorithms now include non-invasive imaging cardiology guidelines and clinical practice in Japan, approaches to risk assessment require updating, and medical care should be appropriately personalized for patients.

## Data Availability

The data associated with the paper are not publicly available but can be partly available from the corresponding author on reasonable request.

## Abbreviations

CAD: Coronary artery disease
CAG: Coronary angiography
CCTA: Coronary computed tomography angiography
CKD: Chronic kidney disease
eGFR: Estimated glomerular filtration rate
FFR: Fractional flow reserve
HF: Heart failure
J-ACCESS: Japanese assessment of cardiac events and survival study by quantitative gated SPECT
JROAD: Japanese registry of all cardiac and vascular diseases
JROADHF: Japanese registry of acute decompensated heart failure
MPI: Myocardial perfusion imaging
OMT: Optimal medical therapy
PCI: Percutaneous coronary intervention
SPECT: Single-photon emission computed tomography
SSS: Summed stress scores
